# Small bowel carcinoid tumor causing intestinal ischemia: A case report with review of the literature^☆^

**DOI:** 10.1016/j.radcr.2022.07.011

**Published:** 2022-07-27

**Authors:** Farid Aassouani, Ayoub Ettabyaoui, Khadija Hinaje, Mohamed Oussama Bahri, Nizar El Bouardi, Karima Oualla, Meryem Haloua, Badreeddine Alami, El Bachir Benjelloun, Nadia Ismaili Alaoui, Meriem Boubbou, Mustapha Maâroufi, My Youssef Alaoui Lamrani

**Affiliations:** a Department of Radiology and Interventional Imaging, CHU Hassan II, FEZ, Sidi Mohammed Ben Abdellah University, Morocco; b Department of Medical Oncology, CHU Hassan II, FEZ, Sidi Mohammed Ben Abdellah University, Morocco; c Department of Visceral Surgery, CHU Hassan II, FEZ, Sidi Mohammed Ben Abdellah University, Morocco; d Department of Nuclear Medicine, CHU Hassan II, FEZ, Sidi Mohammed Ben Abdellah University, Morocco

**Keywords:** Carcinoid, Small bowel, Neuroendocrine tumors, CT, Computerized Tomography, CE CT, Contrast-enhanced computerized tomography, APUD, Amine Precursor Uptake and Decarboxylase, BP, Blood Pressure

## Abstract

*Background:* Intestinal carcinoid tumors are well-differentiated neuroendocrine tumors that are capable of secreting bioactive hormones and/or amines; These tumors are uncommon but are the most common primary tumors of the small intestine. *Case presentation:* We report the case of an 80-year-old woman who presented with a long history (about 14 years ago) of atypical digestive symptoms such as vague abdominal pain, alternating diarrhea, and constipation, treated as functional colopathy without improvement, until the day when she presented with worsening pain that prompted her consultation. CT scan revealed typical manifestations of a carcinoid tumor associated with signs of subacute small bowel ischemia. Despite the surgery being considered a gold standard treatment, it was rejected due to the extent of tumor mesenteric involvement, therefore, the patient received only somatostatin treatment. *Conclusion:* Small bowel carcinoid tumors are rare, with typical imaging features based on cross-sectional imaging (CE CT/MRI). Intestinal ischemia is a well-known complication that can be a factor in mortality.

## Introduction

Carcinoid tumors are a slow-growing type of neuroendocrine tumor that arise from endocrine Amine Precursor Uptake and Decarboxylase: « APUD cells » that can be found throughout the gastrointestinal tract as well as other organs (eg, lung, larynx). In general, they are slow-growing tumors but are nevertheless capable of metastasizing [1].

The most common symptom associated with small bowel carcinoid tumors is atypical abdominal pain, with diagnosis occurring either incidentally or late in the course of illness, when the lesion may manifest as a complication of local mechanical effects or as a result of significant hormone production [2].

Our case focuses on an ileal carcinoid tumor causing subacute intestinal ischemia, which was diagnosed based on cross-sectional and nuclear imaging.

## Case presentation

An 80-year-old patient without medical or surgical history, suffering for about 14 years ago from the occurrence of diffuse and intermittent abdominal pain of moderate intensity associated with transit disorders (alternating diarrhea/constipation). She also complained of weight loss and asthenia during recent years. These symptoms have been taken and treated as functional colopathy without improvement. Furthermore, the patient did not report the notion of secretory diarrhea, flush, or other associated endocrine signs.

Four months before admission, she experienced worsening of abdominal pain with a feeling of heaviness in the left iliac fossa, which led to her consultation. On physical examination, the temperature was 36.2 (oral), BP: 140/70, the pulse rate: 82, and respiratory rate: 17, palpation of the abdomen revealed a palpable mass with a stiff consistency, and tenderness, in the left lower quadrant.

The dosage of serum chromogranin A and 5-hydroxy-indol acetic acid were positive with Chromogranin A: 277 ng/mL (normal range: 27-94), 5-hydroxy-indol acetic acid: 127 μmol/24 h (normal <9.5), the rest of the laboratory findings including WCC, hemoglobin level, platelet count, c reactive protein, liver function tests, serum electrolytes, and urine analysis were normal.

**Fig. 1:**
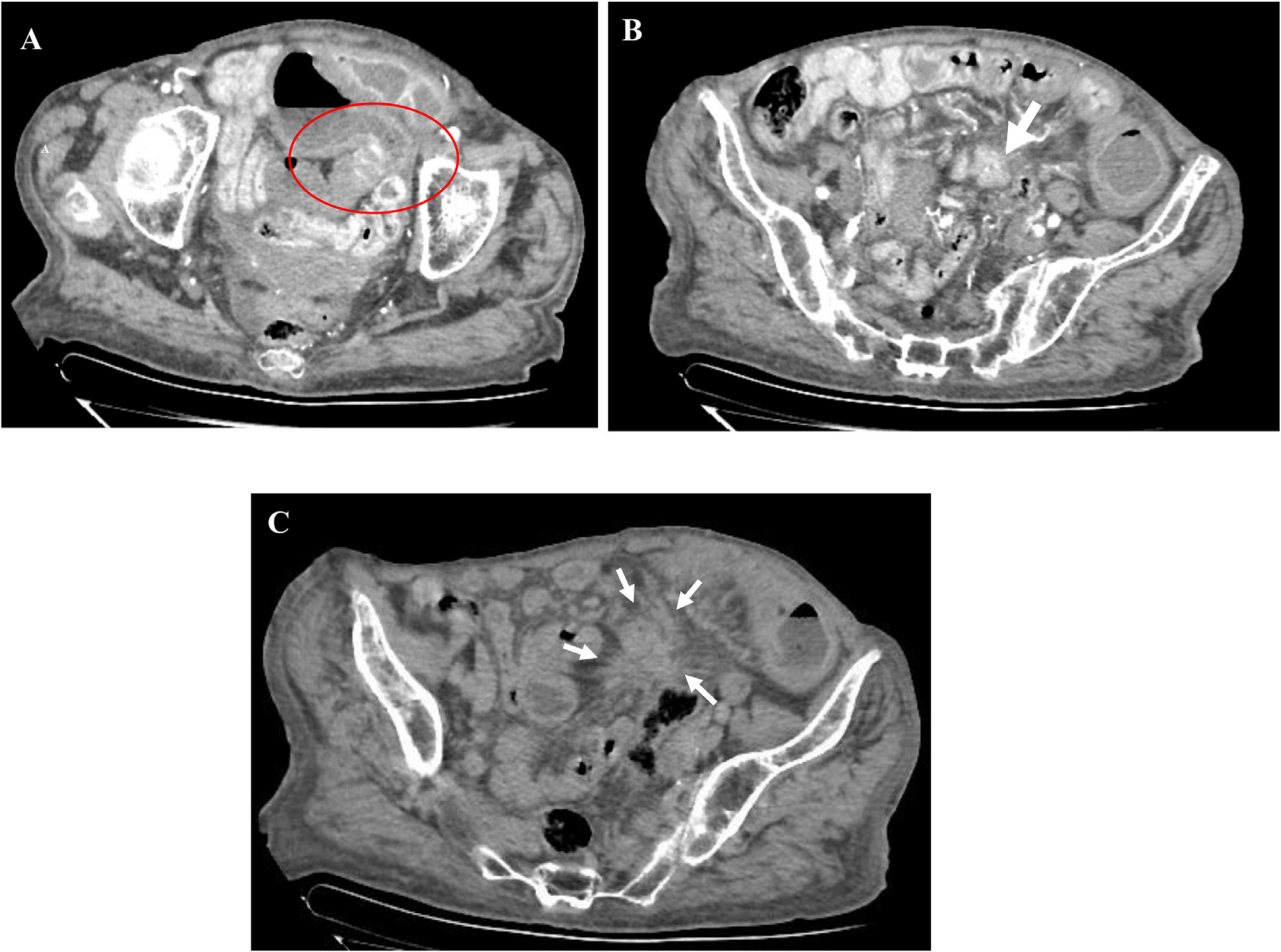
Axial enhanced abdominal CT scan: A, B: arterial phase, C: Portal phase, objectiving: (A) Endoluminal polypoid hypervascular lesion (red circle). (B) Hypervascular mesenteric retractil mass (arrow). (C) Spiculations with stranding bands around the mesenteric mass (arrows)

**Fig. 2:**
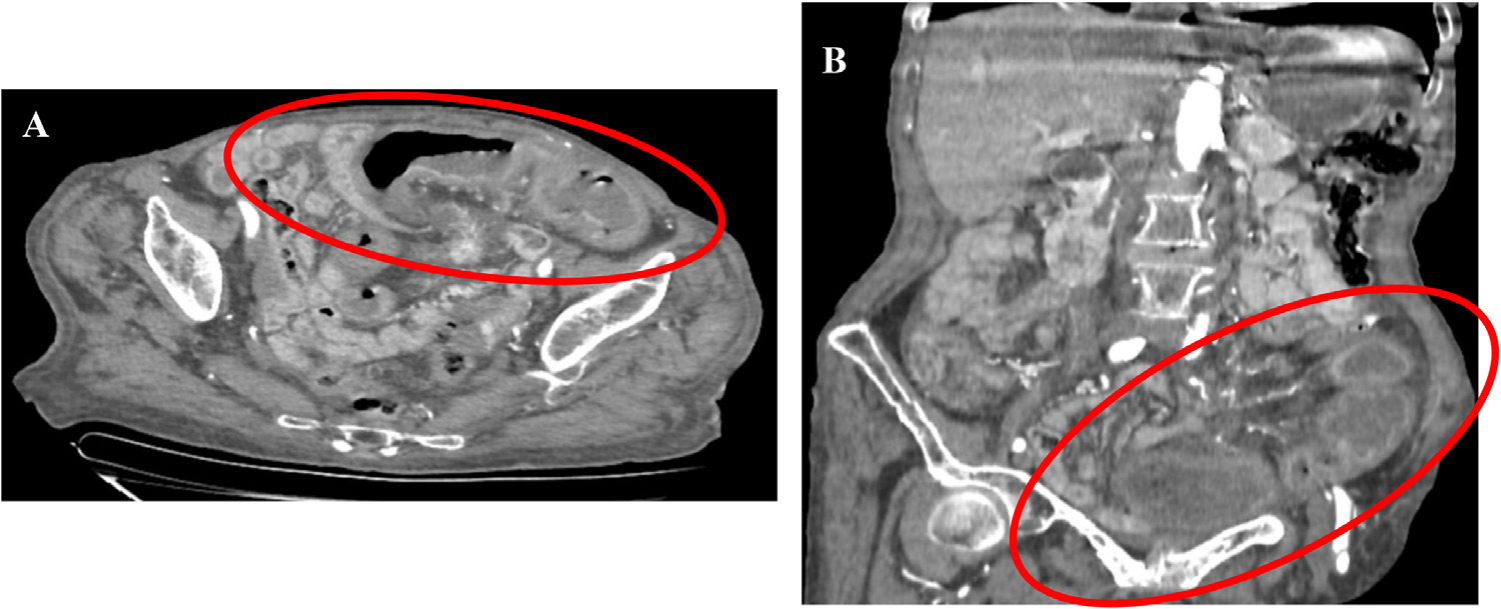
Axial enhanced abdominal CT scan (A) and coronal reformation (B) showing imaging features of subacute intestinal ischemia (red circle).

A triphasic contrast-enhanced abdominal CT scan was performed revealing typical imaging features of an ilial carcinoid tumor of the left iliac fossa wish metastasized in the mesentery (Fig. 1), associated with signs of subacute intestinal ischemia (Fig.2) including mural thickening, lack of enhancement of surrounding bowel loops, and a small amount of ascites. There was no suspect lesion in the liver.

**Fig. 3:**
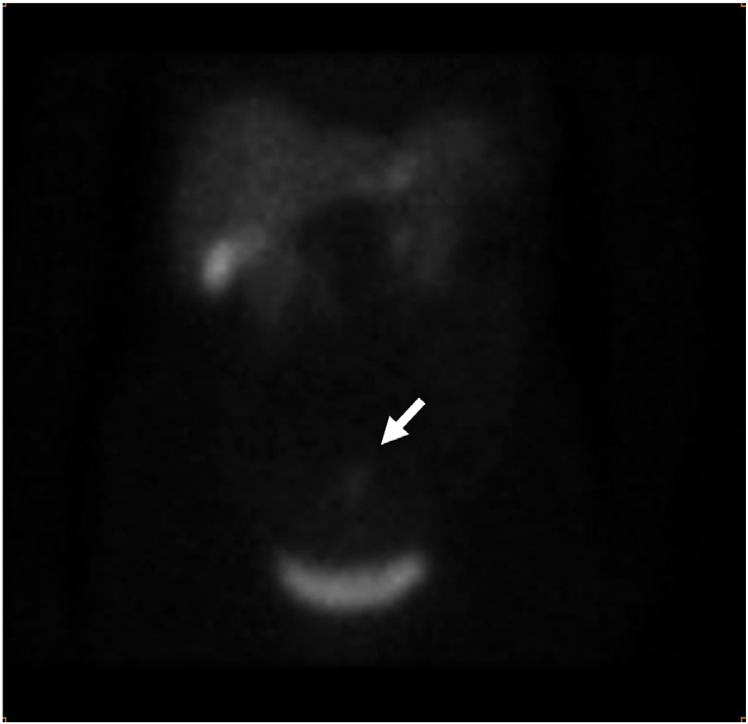
Octreotide scan objectiving uptake of the radiotracer at the level of hypogastrium (arrow) of the radio in hypogastrium.

Somatostatin receptor scintigraphy (also known as octreotide scan) was performed, revealing radiotracer uptake at the level of hypogastrium corresponding to the carcinoid tumor (Fig. 3). Despite the biopsy was not performed due to the absence of an accessible lesion, typical imaging findings, and nuclear imaging results were sufficient to make a certain diagnosis.

Unfortunately, the patient was not eligible for a surgery intervention plan given the extent of mesenteric involvement making the surgical procedure a delicate one, which can impact the patient's median life expectancy, hence, she only received medical treatment based on somatostatin analogs, and remained stable for 2 years.

## Discussion

Small bowel carcinoid tumors are neuroendocrine tumors arising from secretory cells of the neuroendocrine system, they are the most common gastrointestinal tumors and most frequently involve the ileum. [1].

The term “carcinoid” was introduced in 1902 by OBERNDORFER to designate malignant tumors of the small intestine which form metastases but are distinguished from other malignant tumors by slow tumor growth (Oberndorfer, 1907) [2].

The most recent nomenclature that was adopted by the World Health Organization (WHO) uses tumor differentiation as the primary criteria for the malignant potential of neuroendocrine tumors. This allows differentiating well-differentiated tumors of the gastrointestinal tract from poorly differentiated neoplasms [3]. About 99% of all neuroendocrine tumors of the small intestine fall into the more favorable category of well-differentiated tumors [3]. These types of tumors are characterized by excessive production of peptides, neuroamines, and other vasoactive substances. Hence, they can be classified into ‘functioning’ and ‘non-functioning’ types based on their hormone production. The resultant “carcinoid syndrome” caused by excess hormone secretion leads to a variety of symptoms including facial flushing, diarrhea, bronchial constriction, and right‐sided valvular heart disease. Our patient did not experience any of these manifestations.

Non-hormonal symptoms are most often secondary to partial mechanical obstruction of the small bowel, revealed as intermittent vague abdominal symptoms [4]. Other non-hormonal symptoms include anorexia, weight loss, fatigue, and occasionally a palpable abdominal mass.

## Imaging features

Carcinoid tumor diagnosis is based on cross-sectional imaging, in particular contrast-enhanced CT scan that typically reveals a hypervascular mass of variable size, with often radiating borders. Calcification may also be seen in the tumor. Mesenteric metastases may appear sharply defined or spiculated on CT, with bands due to fibrosis and desmoplastic reaction [5]. In the liver, the metastases are highly enhanced in the arterial phase (25-35 seconds) due to their vascularization and become isodense compared to the normal liver parenchyma in the late phase [5].

Nuclear imaging has a crucial role in the detection of neuroendocrine tumors, including carcinoids with somatostatin-binding sites. Several studies have shown that Somatostatin-receptor scintigraphy is a sensitive and noninvasive technique for imaging primary carcinoid tumors and metastatic spread [6]. The diagnosis is also based on immunohistochemical analyses of the tumor. Our patient had a stain for expression of chromogranin A, which was positive. The ki-67 index is a marker for the proliferative activity of the malignancy itself. An index <2% is consistent with low‐grade activity [3].

Small bowel ischemia is a rare complication attributed to hormonal substances produced by the tumor which results in a high mortality rate. In 1961, Moertel was one of the first to report bowel ischemia in four carcinoids (Mortel et al 1961) [7]. Ten years later Anthony and Drury (1970) described elastic vascular sclerosis as a morphologic substrate for this disorder [7]. Because of its rarity, intestinal ischemia is not always considered the primary cause of abdominal pain; mesenteric fibrosis, nodular involvement, progressive tumor growth, and peritoneal adhesions are other well-known complicating factors.

The principal management approach in non-metastatic carcinoid tumors is surgical resection which represents the only curative option [8]. Other possibilities for symptomatic management of patients with hormonal symptoms include biotherapy using drugs such as somatostatin analogs, which have been shown to not only improve symptoms in 70%-80% of cases but also stabilize tumor growth [8].

## Conclusion

Carcinoid tumors are rare tumors of the gastrointestinal tract, however, are the most common primary tumors of the small intestine. Most of these tumors have a very indolent course and may present with nonspecific symptoms.

Imaging features are typical and based on cross-section imaging. Prognosis can be dramatically improved with early diagnosis, and surgical management at this stage is often curative.

The complication to fear during the disease is intestinal ischemia, which often leads the patient to consult an emergency department, and can be a factor in mortality.

## Availability of data and materials

The data sets are generated on the data system of the CHU Hassan II of Fes, including the biological data and the interventional report.

## Author's contribution

**Aassouani farid** is the corresponding author, he participated in the organization and writing of the article and studied the cases with GS. Professor MY.AL, BA, NE, and MH supervised working and validated the figures. Dr. KH and Professor KO have follow-up care for the patient in the medical oncology department Dr. MB and Professor EB have follow-up care for the patient in the surgery department. Professor and chief of the department of radiology MM and MB read and allowed the article for publication. All authors read and approved the final manuscript.
